# Exploring Gaze Dynamics in Virtual Reality through Multiscale Entropy Analysis

**DOI:** 10.3390/s24061781

**Published:** 2024-03-10

**Authors:** Sahar Zandi, Gregory Luhan

**Affiliations:** Department of Architecture, Texas A&M University, College Station, TX 77843, USA; gregory.luhan@tamu.edu

**Keywords:** virtual reality, time series analysis, eye movements, multiscale entropy, user experience, human sensing

## Abstract

This study employs Multiscale Entropy (MSE) to analyze 5020 binocular eye movement recordings from 407 college-aged participants, as part of the GazeBaseVR dataset, across various virtual reality (VR) tasks to understand the complexity of user interactions. By evaluating the vertical and horizontal components of eye movements across tasks such as vergence, smooth pursuit, video viewing, reading, and random saccade, collected at 250 Hz using an ET-enabled VR headset, this research provides insights into the predictability and complexity of gaze patterns. Participants were recorded up to six times over a 26-month period, offering a longitudinal perspective on eye movement behavior in VR. MSE’s application in this context aims to offer a deeper understanding of user behavior in VR, highlighting potential avenues for interface optimization and user experience enhancement. The results suggest that MSE can be a valuable tool in creating more intuitive and immersive VR environments by adapting to users’ gaze behaviors. This paper discusses the implications of these findings for the future of VR technology development, emphasizing the need for intuitive design and the potential for MSE to contribute to more personalized and comfortable VR experiences.

## 1. Introduction

In the changing world of innovation, augmented reality (AR) and virtual reality (VR) technologies are making a powerful impact. They are revolutionizing interaction, visualization, and immersion in industries. These technologies have the ability to transform entertainment by creating gaming experiences and interactive media. They also have the potential to revolutionize education and training through simulated environments. Moreover, they are enhancing care through therapy and reshaping the retail industry with interactive shopping experiences. At the heart of these technologies lies eye tracking, a method that captures a user’s gaze within a virtual environment. By understanding and responding to where users look, eye tracking technology significantly improves interactivity and personalization in AR/VR experiences. This leads to engaging and effective user experiences [1,2,3,4,5,6].

In the realm of healthcare, VR technologies have shown promise in enhancing surgical planning and patient outcomes. For instance, Innocente et al. demonstrated the application of Mixed Reality for Total Hip Arthroplasty Assessment, offering surgeons advanced visualization tools to evaluate and predict post-operative results [7]. This application exemplifies the transformative potential of VR and AR in medical procedures, aligning with our findings on the impact of VR on user experience. Similarly, Su et al. (2022) discuss the use of mixed-reality technology in total knee arthroplasty, highlighting its advantages in complex surgical procedures [8]. Furthermore, Zavala-González et al. (2022) conducted a randomized controlled trial on the effectiveness of adding virtual reality to physiotherapeutic treatment in patients with total hip arthroplasty, showcasing the potential of virtual reality in enhancing rehabilitation outcomes [9]. These studies exemplify the diverse applications of technology in orthopedic surgery and rehabilitation, emphasizing the role of innovative solutions in improving patient care and outcomes.

However despite the progress and widespread acceptance of AR/VR technologies, there are still obstacles that need to be overcome in order to fully utilize their potential and ensure sustainable growth. One major concern in the realm of VR is user comfort. Extended exposure to VR environments can cause discomfort and sensory overload commonly referred to as VR sickness. This condition is characterized by symptoms such as nausea, headaches, and disorientation. Addressing this challenge highlights the importance of creating VR experiences that are not immersive but also comfortable and safe, for users [10,11,12,13,14,15,16].

Maintaining user engagement poses another challenge in virtual reality (VR) applications. As the initial excitement of VR diminishes, it becomes increasingly crucial to sustain user interest and captivation over time. This challenge underscores the importance of creating adaptive content and interface designs that cater to user preferences and behaviors, ensuring a continuous level of engagement and immersion. Furthermore, optimizing interface design for seamless navigation and interaction is essential to provide users with an enjoyable VR experience. Recent works, such as the study by Cannavò et al. [17] on the automatic generation of affective 3D virtual environments from 2D images, highlight the significance of design considerations in retaining users and determining the success of VR applications [18,19,20,21].

Industry leaders such as Meta (previously known as Facebook) along with their Oculus VR systems, Netflix, and Amazon are at the forefront of advancing VR experiences. One of the challenges in this field is finding a balance between the costs involved in creating avatars that enable a sense of embodiment and the need for hardware to track and render natural movements. Meta specifically needs to strike a balance between achieving convincing motion and visual accuracy to maintain a feeling of presence while ensuring accessibility and scalability across their VR platforms, for consumers. Recent research also emphasizes the significance of understanding user behavior eye movement in order to create user-centric VR experiences [22,23,24].

To address these challenges, our study adopts Multiscale Entropy (MSE) analysis, a computational technique initially developed by Javaherian et al. [25]. MSE analysis offers a unique perspective on time series data by dissecting it into non-overlapping windows, revealing patterns and correlations at various scales. Applied to the eye movement data obtained from the GazeBaseVR dataset [26,27], which provides a comprehensive and longitudinal perspective on eye movement behavior in various VR tasks and stimuli, MSE analysis enables us to explore the intricate dynamics of eye movement patterns. This dataset captures the eye movements of participants engaged in a series of diverse and dynamic tasks, including vergence, smooth pursuit, video viewing, self-paced reading, and random saccades, collectively referred to as ‘VRG’, ‘PUR’, ‘VID’, ‘TEX’, and ‘RAN’. These tasks encompass a wide range of stimuli and activities typical in VR environments, making the dataset an invaluable resource for our study [28,29].

In our research, we specifically focus on the horizontal ($\theta_{H}$) and vertical ($\theta_{V}$) components of eye movement, quantified in degrees of visual angle (dva). These components are essential as they provide insights into the intricate dynamics of eye movements in VR. The utilization of MSE analysis allows us to quantify the complexity of these eye movement patterns, offering valuable insights into their predictability and dynamics across different VR tasks and stimuli [26].

By using Mean Squared Error (MSE) on this dataset our study aims to uncover the aspects of eye movements, in different tasks and stimuli. This will help us understand how users interact with and respond to virtual reality (VR) environments. These valuable insights will play a role in addressing the challenges mentioned earlier such as user comfort, engagement, and optimizing interface design. Moreover, they have the potential to drive advancements by allowing us to explore MSE analysis findings for informing VR interface and content design resulting in intuitive, engaging, and comfortable user experiences. Additionally, our research strives to provide suggestions and insights for leaders, in the tech industry. This will assist them in overcoming challenges related to VR production and delivery aligning with the needs and goals of these companies while contributing to the advancement and refinement of VR technologies.

In conducting this study, the paper enhances understanding of eye movement behavior in virtual reality environments and provides practical insights that drive innovation and improvement in the VR industry. The subsequent sections detail the methodology of Multiscale Entropy (MSE) analysis, present findings from this approach, and discuss implications for VR design and user experience. Specifically, the Methodology section outlines the dataset used and the steps taken in the MSE analysis. The Results section presents the complexity patterns observed in eye movements across different VR tasks. The Discussion interprets these findings in the context of current challenges faced by leaders in the tech industry, offering strategic insights and recommendations. Finally, the Conclusion and Future Work summarize the study’s key contributions and propose directions for further research, emphasizing the potential for collaborative efforts with the tech industry.

## 2. Methodology

### 2.1. Dataset Description

The dataset utilized in this study, referred to as GazeBaseVR, is a large-scale longitudinal binocular eye-tracking dataset collected at a high frequency of 250 Hz using an eye-tracking enabled virtual reality (VR) headset. For an in-depth description of the dataset, including the VR application used and the purpose of data collection, readers are referred to Lohr et al. (2023) [26]. The GazeBaseVR dataset includes 5020 binocular recordings from a diverse population of 407 college-aged participants, recorded up to six times each over a 26-month period. Each session included a series of five different eye-tracking (ET) tasks designed to elicit various eye movements and behaviors. These tasks were a vergence task, horizontal smooth pursuit task, video-viewing task, self-paced reading task, and random oblique saccade task. These tasks were selected to simulate a wide range of visual experiences in VR, from focusing on specific objects at varying depths to engaging in activities that mimic everyday visual behaviors, providing a comprehensive assessment of eye movement patterns. This comprehensive set of tasks was carefully selected to cover a broad spectrum of eye movements and to provide insights into both the micro-movements and overall behavioral patterns of the participants’ eye movements in a virtual reality environment.

The dataset’s longitudinal nature, with multiple recordings of the same individuals over an extended period, provides a unique opportunity to investigate not just the variability and commonalities in eye movements across individuals and tasks but also changes and trends over time, whether due to learning, adaptation, or other long-term factors.

Furthermore, the diversity of the participant pool in terms of demographics and the detailed recording of participant details make this dataset particularly valuable for investigating factors such as the impact of demographic variables on eye movement behavior and the potential for personalized or adaptive VR experiences based on eye movement patterns.

From a technical perspective, the data were captured using SensoMotoric Instrument’s eye-tracking virtual reality head-mounted display (ET-HMD) based on the HTC Vive. This device tracks both eyes at a nominal sampling rate of 250 Hz with a high spatial accuracy and precision, ensuring detailed and reliable eye movement data. The dataset provides an extensive range of metrics, including gaze direction vectors and positional data, which can be used to derive various eye movement characteristics.

In our study, we specifically focus on the horizontal (*x*) and vertical (*y*) components of the eye rotation, denoted as $\theta_{H}$ and $\theta_{V}$, respectively, in terms of degrees of visual angle (dva). These components are crucial for understanding the direction and extent of eye movements, especially in the context of interactive VR environments where users are constantly engaging with dynamic content. The conversion of the direction vector v=[x,y,z] into these components is performed using the following equations:

(1) $$\theta_{H}=\frac{180}{\pi }\arctan2\left(x,\sqrt{y^{2}+z^{2}}\right)$$

(2) $$\theta_{V}=\frac{180}{\pi }\arctan2\left(y,z\right)$$

The “*z*” component in this vector represents the depth axis in a three-dimensional coordinate system, indicating the depth at which a user is looking into the screen or virtual environment. This depth component is essential for calculating the exact direction of gaze in the 3D space of VR, providing a comprehensive understanding of eye movement behavior.

These equations provide a standardized way of quantifying eye movement in terms of horizontal and vertical rotation angles, facilitating the analysis of eye movement patterns and the application of Multiscale Entropy (MSE) analysis to assess the complexity and predictability of eye movements across different VR tasks [26,27].

### 2.2. Analysis of Multiscale Entropy

A system’s time series data can be affected by various noises that stem from its interaction with the surrounding environment. These noises can introduce short-term correlations that falsely appear as long-term effects in the time-series analysis. Therefore, it is crucial to mitigate the impact of these noises and the associated short-term correlations. This is achieved through a process known as coarse graining. In this process, a time series with *N* data points ($y_{1},y_{2},\ldots ,y_{N}$) is divided into equal-length, non-overlapping segments called windows, each of length λ. Within each window, the data points are averaged to produce a new time series with reduced granularity as follows:

(3) $$y_{p}^{\left(\lambda \right)}=\frac{1}{\lambda }\sum_{q=1}^{\lambda }y_{(p-1)\lambda +q}$$

This results in a new series composed of the averaged data points $\left(y_{1}^{\left(\lambda \right)},\ldots ,y_{N_{\lambda }}^{\left(\lambda \right)}\right)$, where λ serves as the scaling factor. The restructured data can then be represented by vectors of *m* dimensions as shown below:

(4) $$Y_{m}^{\left(\lambda \right)}\left(p\right)=\left(y_{p}^{\left(\lambda \right)},\ldots ,y_{p+m-1}^{\left(\lambda \right)}\right)$$

The subsequent step involves counting the number of vector pairs whose mutual distance is less than a specified value *r*. This quantity is denoted as $n_{m}\left(r,\lambda \right)$. This counting is also performed for vectors of dimension m+1, denoted as $n_{m+1}\left(r,\lambda \right)$. The measure of complexity, known as sample entropy, is then computed with the following formula:

(5) $$S_{e}\left(m,r,\lambda \right)=-\log\left(\frac{n_{m+1}\left(r,\lambda \right)}{n_{m}\left(r,\lambda \right)}\right).$$

Given that $n_{m+1}\left(r,\lambda \right)\leq n_{m}\left(r,\lambda \right)$, it follows that $S_{e}$ is non-negative. A graph plotting sample entropy against the scale factor illustrates the correlation ranges present in the time series. This comprehensive procedure is referred to as multiscale entropy (MSE) analysis [25].

### 2.3. Computational Methodology

The Multiscale Entropy (MSE) analysis of the eye movement data was conducted using a Python script that leverages libraries such as NumPy for numerical operations and Matplotlib for visualizing the entropy trends. The script’s core consists of functions specifically designed to perform coarse graining, calculate sample entropy, and compile multiscale entropy across varying scales for a comprehensive understanding of complexity in eye movement data. The choice of Python and these libraries was driven by their robustness, efficiency, and widespread use in scientific computing, ensuring reliability and reproducibility of our results.

**Figure 1:**
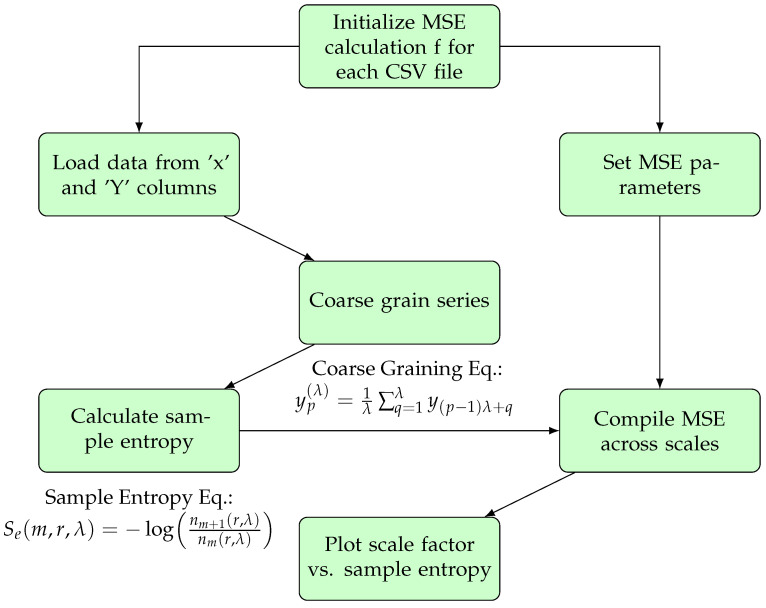
Flowchart illustrating the MSE calculation process with included equations.

As illustrated in Figure 1, the computational process of Multiscale Entropy (MSE) analysis involves several key steps. Starting with the initialization of the process for each file in the dataset, it proceeds with loading the data specifically from the ‘*x*’ and ‘*y*’ columns indicative of the horizontal and vertical gaze positions. Parameters for the MSE analysis are set according to standard practices and preliminary analysis to ensure robustness and relevance of the results. The time series data are then coarse grained at multiple scales, upon which sample entropy is calculated to quantify the complexity at each scale. Finally, these complexity measures are compiled across all scales to visualize and analyze the variation of eye movement complexity. The coarse graining and sample entropy calculations are guided by specific equations, integral to understanding the underlying mechanics of this analysis.

### 2.4. Rationale behind Parameters and Approach

The selection of parameters for Multiscale Entropy (MSE) analysis, particularly the max_scale parameter set to 20, is instrumental in dissecting the complexity and predictability of eye movements within virtual reality (VR) environments. This value is chosen based on a balance between capturing a broad range of temporal scales and maintaining computational efficiency. A max_scale of 20 typically covers the spectrum from rapid, short-term dynamics to more extended patterns of engagement without overly smoothing the data, thus preserving the detailed behavior inherent in the eye movement recordings. This careful calibration allows for a nuanced examination of eye movement behavior across these scales, revealing insights into immediate reactions and prolonged engagement patterns. The choice is further supported by empirical testing and alignment with standard practices in time-series analysis, ensuring that the scale range is both practical and effective for capturing the complexities of eye movement in VR environments.

**Interpreting MSE Analysis:** Interpretations of MSE plots draw upon complex signal analyses, aligning with methodologies used in various scientific studies. These interpretations help us understand the diverse nature of eye movements, from short-term adjustments to long-term strategies [30,31,32,33].

- **Rising Trends (Increasing Sample Entropy with Scale Factor):** Gradual increases in sample entropy across scales can indicate adaptive or exploratory behavior, reflecting users’ continuous engagement and adjustment to the complexities of the VR environment. This pattern may denote a dynamic and enriching interaction, where the gaze behavior becomes progressively complex and unpredictable, suggesting deepening engagement or increasing task difficulty.
- **Fluctuating Trends (Variable Sample Entropy):** Fluctuations in sample entropy highlight moments of transition in cognitive demand or strategy. Peaks in entropy may indicate periods of heightened complexity or adaptation to new stimuli, whereas valleys suggest more predictable or habitual gaze patterns. Such variability is crucial for understanding how users interact with changing content or complexities within the VR environment.
- **Steady Trends (Consistent Sample Entropy across Scale Factors):** A consistent level of sample entropy across scales suggests a uniform cognitive load or a continuous level of interaction complexity. This trend may indicate that users have reached an equilibrium in their gaze behavior, efficiently balancing exploration and exploitation of visual information in the VR environment. It might also suggest that the VR task or experience is consistently engaging users at a stable level throughout.

**Comprehensive Insights from MSE Analysis:** The understanding of these trends provides deeper insights into the cognitive load, user engagement, and possible avenues for VR experience enhancement as follows:

- Datasets with rising trends and fluctuations might indicate a constantly adapting and highly engaged user, with significant moments of cognitive demand interspersed with periods of routine interaction.
- Steady trends can suggest a balanced and consistent level of interaction, possibly pointing towards efficient user navigation or well-calibrated task demands within the VR environment.
- The absence of declining trends in our analysis indicates that users do not typically exhibit decreasing complexity or increasing predictability in their gaze patterns across the studied tasks, highlighting the consistent challenge or engagement presented by the VR tasks.

**Scale Factor and Sample Entropy:** Both the scale factor and sample entropy are pivotal in MSE analysis. The scale factor, a dimensionless number, determines the granularity of the time series analysis, affecting the temporal resolution and interpretability of complexity patterns. Specifically, it represents the level of coarse-graining applied to the time series data, signifying the extent to which data points are averaged together. A scale factor of 1 indicates no coarse-graining, thus reflecting the original data, while higher-scale factors imply a progressive averaging of data points, highlighting broader, long-term patterns.

Sample entropy, also a dimensionless measure, quantifies the regularity and unpredictability within the time series across scales. As a statistical tool, it offers insights into the complexity inherent in the eye movement data. A decrease in entropy at larger scale factors typically implies increasing predictability and regularity over longer periods, while an increase might indicate more complex and less predictable patterns, reflecting changes in cognitive demand or task complexity. Together, these metrics provide a comprehensive understanding of the intricate dynamics of eye movements in VR environments, reflecting the cognitive processes and engagement levels of users.

## 3. Results and Discussion

Understanding the intricacies of eye movement behavior in virtual reality (VR) environments is crucial for enhancing user experience and interface design. This study applies Multiscale Entropy (MSE) analysis to dissect the complexity of eye movements, specifically focusing on the horizontal (*x*) and vertical (*y*) components of gaze position. MSE, a robust method for quantifying the complexity and predictability of time series data, is particularly well suited for exploring the nuanced patterns of eye movement in various VR tasks.

By analyzing these components across different scales, the study reveals insights into the dynamic nature of gaze behavior and its implications for VR interaction. The following subsections present the key findings from the MSE analysis, highlighting the distinct complexity patterns observed in eye movements across a series of VR tasks. Through these results, the study aims to shed light on the multifaceted nature of human gaze in virtual environments and contribute to the optimized design and development of VR systems.

### 3.1. Multiscale Entropy Analysis of Eye Movements in the VRG Task

**Task Context:** The vergence (VRG) task involves eye movements where participants adjust their gaze to focus on objects at varying depths within the virtual environment, simulating a 3D experience. This task is critical for depth perception and fundamental to a realistic and immersive virtual environment.

**Figure 2:**
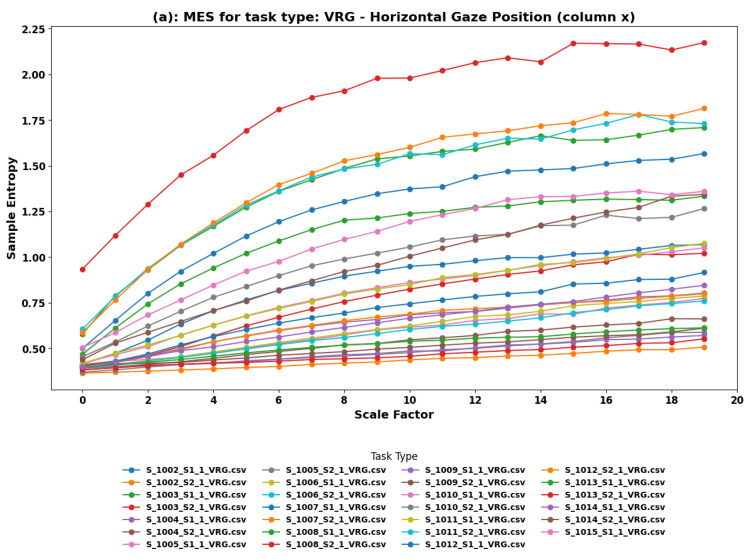

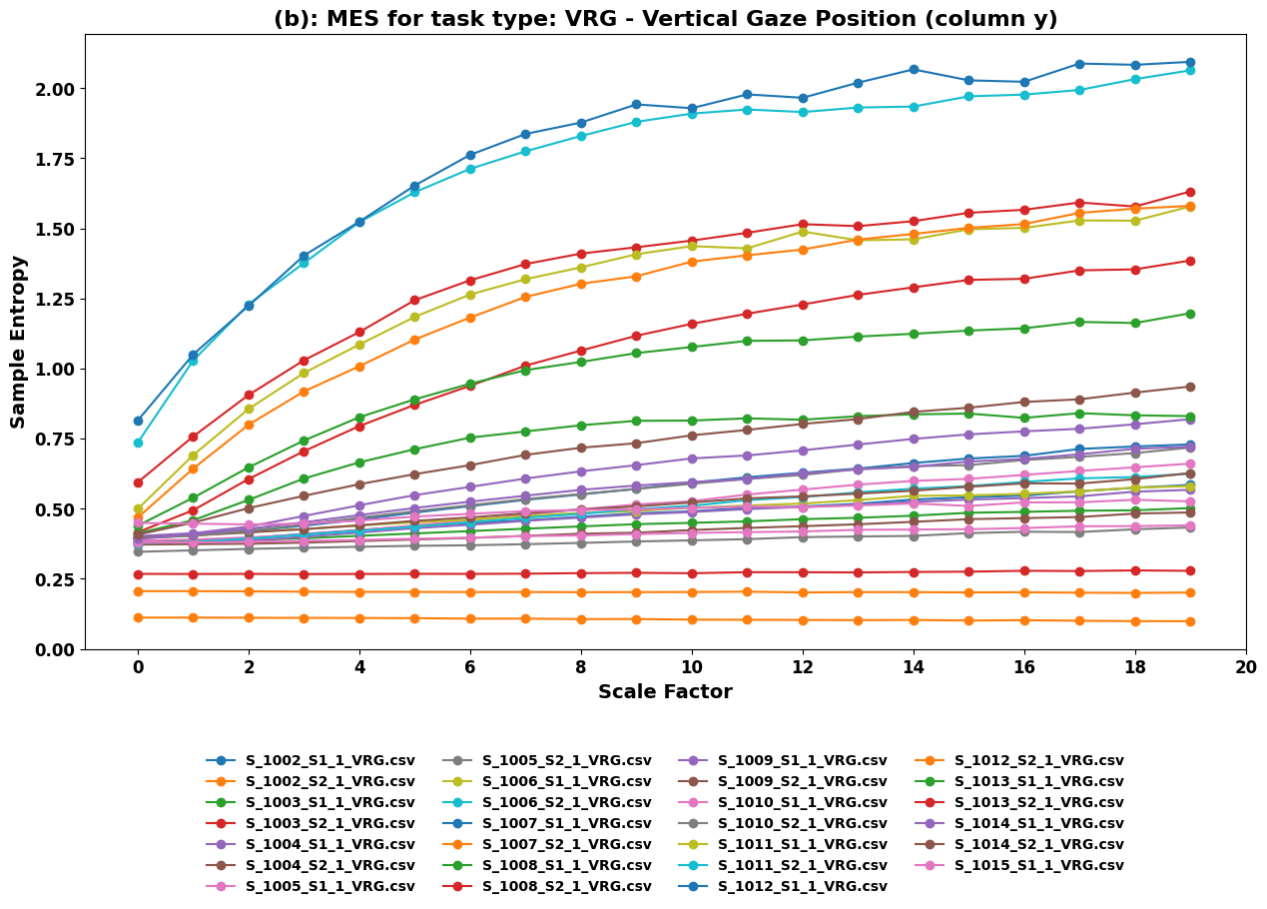
Multiscale Entropy analysis of eye movements in the VRG Task. (**a**) Horizontal and (**b**) Vertical Gaze Position. The VRG task involves vergence eye movements, where participants adjust their gaze to focus on objects at varying depths, simulating a 3D environment in VR.

**MSE Findings:** Multiscale Entropy (MSE) analysis was performed on both the horizontal (*x*) and vertical (*y*) components of eye movements during the VRG task. Figure 2 illustrates the sample entropy across different scale factors for the horizontal and vertical gaze positions, respectively. The sample entropy was plotted across different scale factors for both gaze positions, illustrating the complexity and predictability of eye movements as participants engaged in depth-adjusting tasks.

**Interpretation:**

**Horizontal Component (*****x*****) Analysis:** For the horizontal component, the rising trends in sample entropy across nearly all datasets indicate an increase in complexity and unpredictability of gaze patterns as the scale factor increases. The patterns resembling y=ln(x) suggest an adaptive or exploratory behavior, reflecting continuous engagement and adjustment by the users to the depth variations in the VR environment. The slight positive slope observed in the rest of the datasets, similar to $y=\frac{1}{n}x$, although indicative of a rising trend, suggests a more gradual increase in complexity, possibly due to a more steady or predictable aspect of the vergence task.

**Vertical Component (*****y*****) Analysis:** In the vertical component, the datasets split into two distinct behaviors: approximately half exhibiting a logarithmic increase, indicating adaptive behavior as seen in the horizontal component, and the other half displaying a steady trend, suggesting a uniform cognitive load or a consistent level of interaction complexity. The consistent sample entropy across scales in these steady trend datasets might imply that for certain aspects of the VRG task, users maintain a stable gaze behavior, efficiently balancing exploration and exploitation of depth information presented in the virtual environment.

**Comprehensive Insights from VRG Task Analysis:** The observed MSE trends for the VRG task provide valuable insights into how users interact with and adapt to 3D virtual environments. Rising trends across most datasets, especially in the horizontal component, underscore the dynamic and engaging nature of the task, prompting users to continuously adjust their gaze strategy. The variability in entropy, particularly the distinction between rising and steady trends in the vertical component, highlights the complexity of depth perception and the different strategies users might employ to navigate 3D spaces. These findings emphasize the need for VR content design to consider the varying cognitive loads and interaction complexities inherent in depth-oriented tasks, aiming to optimize user comfort and enhance the overall immersive experience.

**Implications for VR Content Design and User Experience:** Understanding the complexity patterns in eye movements during vergence tasks can inform the design of more intuitive and comfortable VR experiences. By aligning VR content and interface design with the natural tendencies of human ocular activity in 3D environments, developers can create more immersive and engaging applications that cater to the depth perception needs of users. Furthermore, recognizing the adaptive behaviors and stable interaction trends in eye movements can help optimize the balance between visual challenge and user comfort, contributing to the reduction of VR sickness and enhancing long-term user engagement.

### 3.2. Multiscale Entropy Analysis of Eye Movements in the VID Task

**Task Context:** The Video Viewing (VID) task is a central component of virtual reality (VR) experiences, involving participants watching diverse video content. This immersive activity requires users to maintain sustained attention and engagement, as they visually explore and react to dynamic visual scenes. Understanding how gaze behavior adapts during video viewing is crucial for enhancing user experience and optimizing content design in cinematic or narrative VR settings.

**Figure 3:**
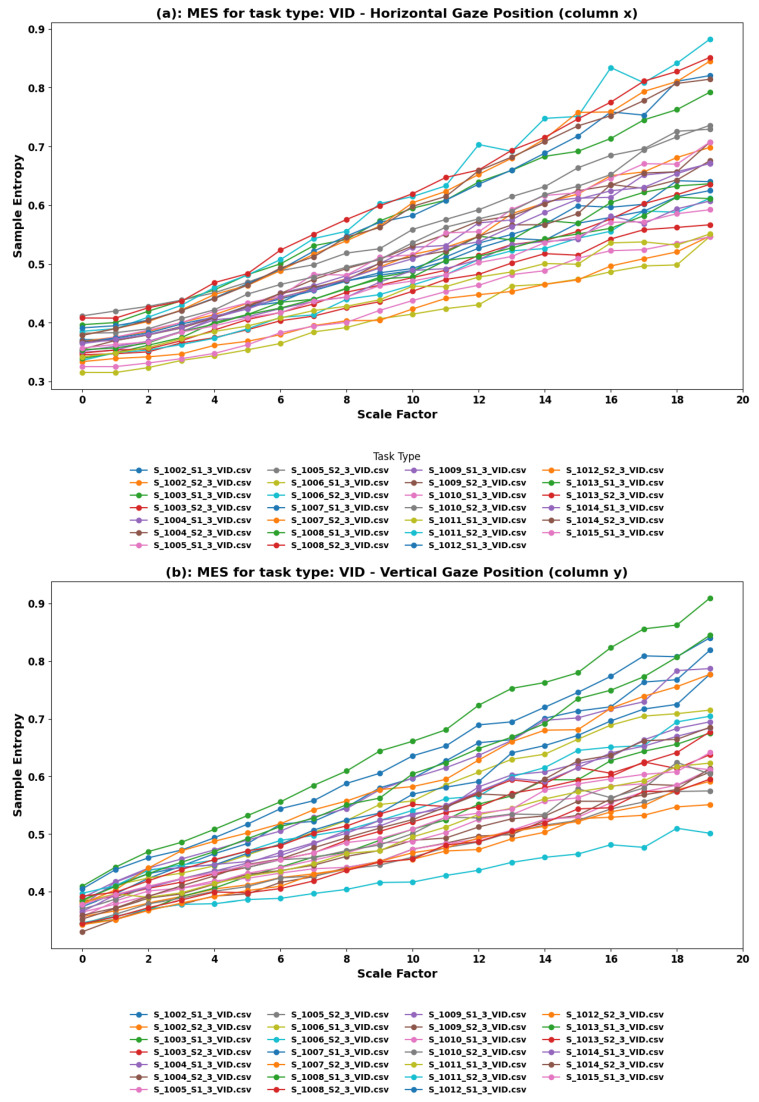
Multiscale Entropy analysis of eye movements in the VID Task. (**a**) Horizontal and (**b**) Vertical Gaze Position. The VID task involves participants watching videos, a common activity in VR, focusing on understanding how gaze behavior changes during passive viewing.

**MSE Findings:** For the VID task, Multiscale Entropy (MSE) analysis was conducted on the horizontal (*x*) and vertical (*y*) components of eye movements. Figure 3 showcases the sample entropy across different scale factors for horizontal and vertical gaze positions, respectively. The analysis produced plots showing sample entropy across various scale factors for both gaze positions, reflecting the complexity and predictability of eye movements as participants interacted with video content.

**Interpretation:**

**Linearly Rising Trends:** Both the horizontal and vertical components exhibit linearly rising trends in sample entropy with different slopes. This consistent increase across the scale factors suggests that as the viewing time extends, the complexity of gaze patterns also increases. The variation in slopes between the horizontal and vertical components could indicate different levels or types of engagement and visual exploration. For instance, steeper slopes may represent periods of more intense visual exploration or engagement with the content, possibly during dynamic or rapidly changing scenes.

**Comprehensive Insights from VID Task Analysis:** The MSE analysis of eye movements during the VID task underscores the dynamic interaction between viewers and video content in VR environments. The linearly rising trends across most datasets highlight the ongoing adaptation and engagement of viewers with the content, providing a quantitative measure of how video narratives and visual stimuli influence gaze behavior. These insights can drive the development of more captivating and immersive VR video experiences, ensuring that content remains engaging and aligned with the viewer’s natural gaze dynamics.

**Implications for Content Design and User Experience:** These findings have significant implications for VR video content creators. Understanding the nature of gaze complexity during video viewing can help in identifying key moments for narrative or visual shifts to maintain or enhance viewer engagement. For example, creators might introduce changes in content at points where the entropy trend suggests a decrease in engagement or predictability, thereby reinvigorating viewer attention and interest.

### 3.3. Multiscale Entropy Analysis of Eye Movements in the PUR Task

**Task Context:** The pursuit (PUR) task is a crucial component in understanding user interaction in virtual reality (VR), particularly focusing on smooth pursuit eye movements. Participants track moving targets, a fundamental activity for simulating realistic scenarios in interactive and gaming environments. This task is vital for examining the mechanisms of visual tracking and how individuals maintain focus on dynamic objects within VR.

**Figure 4:**
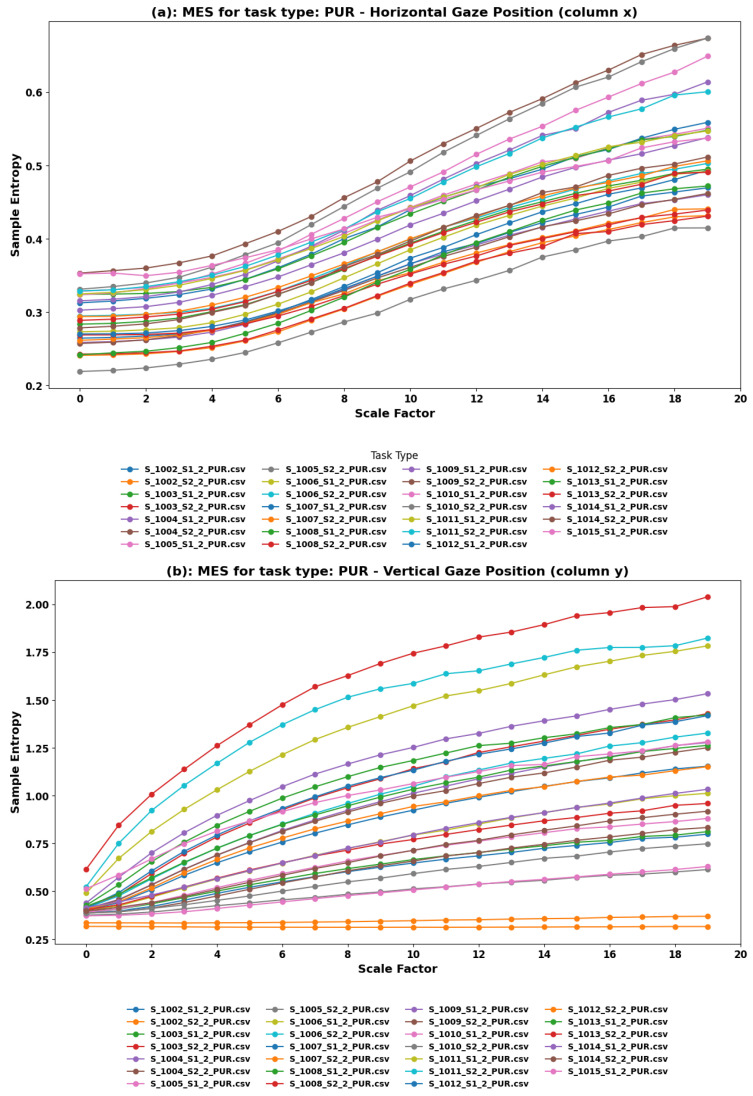
Multiscale Entropy analysis of eye movements in the PUR task. (**a**) Horizontal and (**b**) Vertical Gaze Position. In the PUR task, subjects engage in smooth pursuit movements, following moving objects with their eyes, which is essential for tracking moving stimuli in VR.

**MSE Findings:** Multiscale Entropy (MSE) analysis was performed on both the horizontal (*x*) and vertical (*y*) components of eye movements during the PUR task. Figure 4 illustrates the sample entropy across different scale factors for the horizontal and vertical gaze positions, respectively. The analysis yielded entropy trends across various scale factors, reflecting the complexity and predictability of eye movements as participants engaged in tracking moving targets.

**Interpretation:**

**Horizontal Component—Logistic Function Trends:** The horizontal eye movements exhibited trends similar to logistic functions with varying heights, indicating a saturation effect in the complexity of eye movements.

**Vertical Component—Logarithmic and Steady Trends:** Vertical eye movements predominantly displayed a logarithmic increase in complexity, suggesting a continuous adaptation or increasing challenge in tracking the vertical motion of targets. A few datasets exhibited steady trends, which might indicate consistent tracking behavior or uniform task difficulty across those particular sessions.

**Comprehensive Insights from PUR Task Analysis:** The MSE trends in the PUR task provide a detailed account of how users engage with moving targets in VR. The logistic-like patterns in horizontal movements suggest a bounded complexity, perhaps due to the capabilities of the human visual system or the specific nature of the task. On the other hand, the logarithmic trends in vertical movements reflect a more nuanced adaptation process, with users possibly exerting more effort or strategy in tracking vertical motion.

**Implications for VR Content Design and User Experience:** Understanding these intricate patterns of eye movement can inform the design of VR content, particularly in scenarios requiring object tracking or interaction with moving elements. Designers and developers might consider the limitations and capabilities reflected in the logistic function trends when creating moving targets or interactive elements, ensuring that these are within comfortable tracking ranges for users. Additionally, the varying complexity in vertical tracking might influence how vertical motion is incorporated into VR experiences, balancing challenge and comfort to enhance overall engagement and minimize discomfort or disorientation.

### 3.4. Multiscale Entropy Analysis of Eye Movements in the TEX Task

**Task Context:** The text reading (TEX) task simulates the act of reading within a virtual environment, an essential component in educational and informational VR applications. This task involves participants reading text presented in the VR space, requiring saccadic movements and fixations similar to those in real-world reading scenarios. Understanding the complexity of these eye movements is vital for creating VR systems that support effective and comfortable reading experiences.

**Figure 5:**
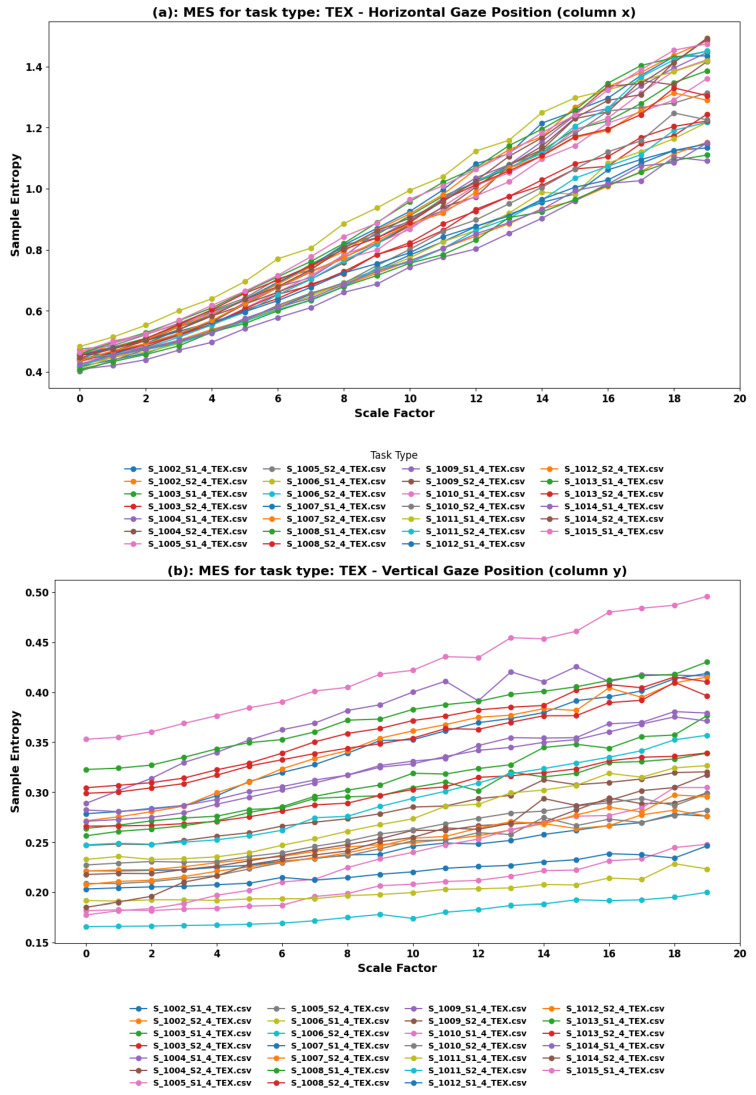
Multiscale Entropy analysis of eye movements in the TEX task. (**a**) Horizontal and (**b**) Vertical Gaze Position. The TEX task represents reading text in VR, a scenario that involves distinct eye movement patterns due to the linear nature of text and frequent line shifts.

**MSE Findings:** In the TEX task, Multiscale Entropy analysis was applied to both the horizontal (x) and vertical (y) components of eye movements associated with reading. The analysis yields distinct patterns of sample entropy across different scale factors for both gaze positions. Figure 5 represents the sample entropy across different scale factors for horizontal and vertical gaze positions, respectively. Horizontally, there is a trend of linearly rising entropy with minor fluctuations, indicative of the systematic left-to-right eye movement typical of reading in many languages. Vertically, most datasets show a rising trend with more pronounced fluctuations, reflecting the varied complexity as eyes move down the page or screen and possibly adjust to different lengths of text or formatting changes.

**Interpretation:** The MSE trends observed in the TEX task shed light on the nuanced complexity of eye movements during VR reading activities. In the horizontal component, the mostly linearly rising trend with little fluctuations suggests a consistent, though slightly increasing, complexity in eye movement as the text progresses, likely due to accumulating cognitive load or adapting strategies for text navigation. The presence of minor fluctuations might also indicate variations in line lengths, word complexity, or punctuation that momentarily alter the reading rhythm.

For the vertical component, the varying slopes and more pronounced fluctuations in rising trends suggest a diverse range of strategies or challenges as users navigate between lines or paragraphs. This variability might reflect differences in text layout, the effort required to adjust to new lines, or the cognitive processing of section breaks or paragraph endings. A few datasets showing a more steady trend might represent users with more uniform reading strategies or perhaps simpler text structures that elicit more consistent eye movement patterns.

In both cases, the MSE analysis illustrates the complex landscape of reading behavior in VR, offering valuable insights for designing VR content and interfaces that accommodate the natural reading process. By aligning text presentation with the observed entropy trends, developers can enhance readability, reduce cognitive load, and improve user comfort and engagement with textual content in VR.

**Implications for VR Content Design and User Experience:** Understanding the entropy patterns in reading-related eye movements enables VR content developers to optimize text layout, pacing, and interaction in educational and informational applications. The insights from the horizontal and vertical components of eye movement can inform decisions about font size, line spacing, and the placement of textual elements to align with natural reading patterns and minimize discomfort or disorientation. Furthermore, recognizing the variability in individual reading strategies, as suggested by the different slopes and fluctuations in entropy, underscores the need for customizable or adaptive reading interfaces that can accommodate a range of user preferences and capabilities.

### 3.5. Multiscale Entropy Analysis of Eye Movements in the RAN Task

**Task Context:** The random saccades (RAN) task captures the eye movements when participants are required to swiftly and randomly shift their gaze between various points within the VR environment. This task simulates the erratic and spontaneous eye movements that occur as users navigate through and respond to unpredictable or rapidly changing virtual scenarios. Assessing the complexity of these movements is essential for understanding user navigational strategies and responsiveness in a dynamic VR landscape.

**Figure 6:**
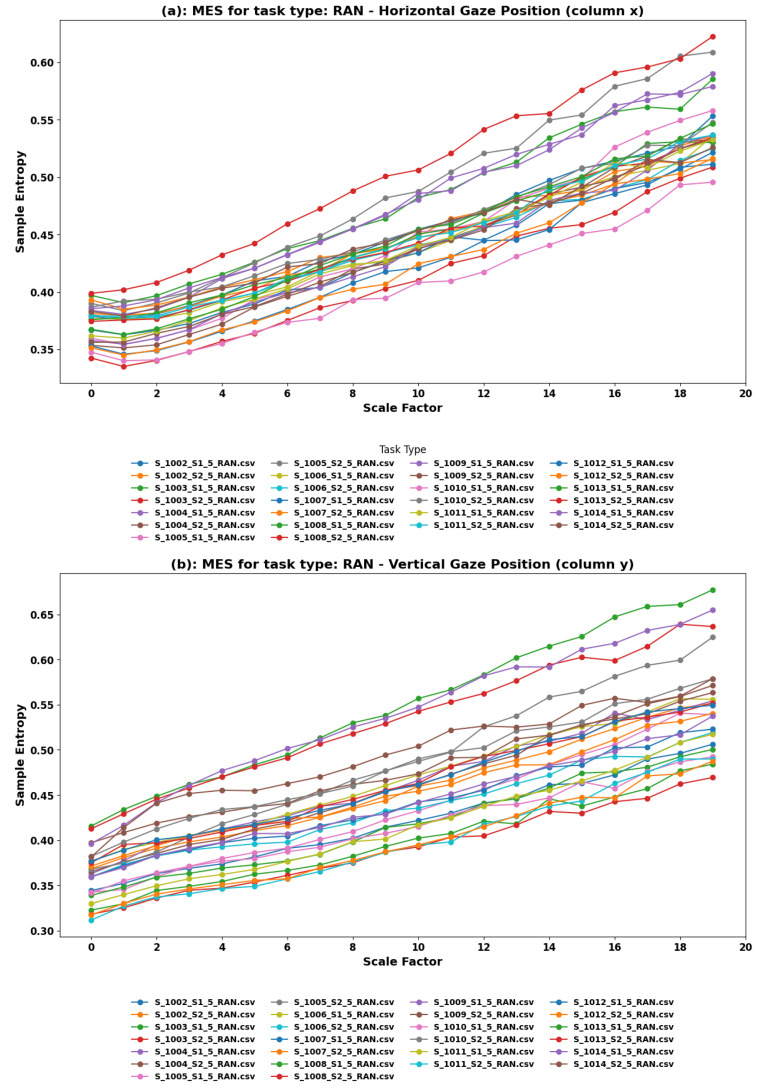
Multiscale Entropy analysis of eye movements in the RAN task. (**a**) Horizontal and (**b**) Vertical Gaze Position. In the RAN task, participants perform random saccades, simulating unpredictable eye movements as they might occur in a dynamic and unstructured VR environment.

**MSE Findings:** Multiscale Entropy analysis applied to the RAN task’s eye movements, both horizontal (x) and vertical (y), shows a pattern of rising entropy, depicted in Figure 6. The entropy progression in both components, characterized by a generally linear rise with similar slopes across different datasets, suggests a consistent increase in complexity as the scale factor increases. This uniformity in the trend across datasets might indicate a standardized response to the random saccades required in the task, reflecting the users’ adaptation to the unpredictability inherent in the activity.

**Interpretation:** The MSE findings from the RAN task offer insights into the nature of eye movements during random, quick shifts of gaze in VR. The consistently rising entropy across scales in both horizontal and vertical components suggests that participants exhibit a gradual increase in complexity in their eye movements, possibly as a reflection of ongoing adaptation to the unpredictability and dynamism of the task. This adaptation might be indicative of users’ efforts to optimize their gaze strategy to efficiently deal with random stimuli, maintaining a level of readiness to shift focus swiftly and accurately.

The similar slopes in the linear rise of entropy among datasets imply a commonality in the approach users take to handle random saccades in VR. This could be due to the nature of the task, which requires a general alertness and readiness to move the gaze randomly, leading to a uniform increase in complexity across users.

**Comprehensive Insights from RAN Task Analysis:** The RAN task’s analysis underscores the importance of understanding spontaneous and random eye movements in VR, as they are indicative of how users might navigate and respond to unstructured or unpredictable environments. Insights from this task can inform the design of VR experiences that require users to be alert and ready to change focus quickly, such as in certain gaming scenarios or training simulations where rapid situational awareness is critical.

**Implications for VR Content Design and User Experience:** Understanding the consistent increase in complexity in eye movements during random saccades tasks can help VR designers create environments that are attuned to the naturalistic movements of users. By recognizing the characteristics of eye movement in unpredictable scenarios, VR experiences can be optimized to accommodate or even leverage these natural behaviors, enhancing user navigation, orientation, and overall engagement with the content. Moreover, insights into the uniform strategies employed by users across different datasets may assist in standardizing certain aspects of VR design, ensuring a coherent and user-friendly experience even in the most dynamic and unstructured virtual scenarios.

### 3.6. Deciphering Eye Movement Dynamics

The MSE analysis reveals diverse patterns in eye movements, reflecting users’ cognitive states and adaptability within VR environments. The absence of declining trends suggests users may be consistently engaged or adapting to the complexities of VR tasks. Rising, fluctuating, and steady trends indicate different user interactions, each providing insights into how participants navigate and respond to VR environments. These patterns highlight the need for adaptive VR systems that are responsive to the intricate behaviors of users.

### 3.7. Insights into User Comfort from MSE Analysis

Our study’s Multiscale Entropy (MSE) analysis of eye movements across various VR tasks provides critical insights that can significantly contribute to enhancing user comfort in VR environments. By examining the complexity of eye movements, we can identify potential triggers of discomfort and devise strategies to mitigate these issues. For instance, tasks that exhibit high complexity in eye movements may signal a higher cognitive load or visual stress, potentially leading to discomfort or VR sickness.

Specifically, the analysis revealed that certain VR tasks, such as the PUR (pursuit) and RAN (random saccades) tasks, show pronounced complexity in eye movement patterns. This complexity could indicate that users are required to constantly adapt their gaze, potentially leading to eye strain or fatigue over extended periods. To address this, VR content designers can consider integrating more natural gaze paths and reducing the frequency of abrupt visual changes in these tasks. Moreover, implementing dynamic difficulty adjustments based on real-time MSE analysis of eye movements can help maintain user engagement without overwhelming them, thereby enhancing comfort.

Furthermore, our findings suggest that steady trends in eye movement complexity, observed in tasks like the TEX (text reading) task, could be leveraged to design VR experiences that align with natural eye movement patterns, minimizing the risk of discomfort. By tailoring content to match these naturalistic patterns, VR experiences can become more intuitive and less taxing on the user, promoting longer and more comfortable usage.

Incorporating adaptive interfaces that respond to the MSE analysis of a user’s eye movements can also offer personalized comfort adjustments. For example, interfaces could automatically adjust text size, brightness, or even the pace of content based on real-time analysis, ensuring a comfortable viewing experience tailored to individual user needs.

Overall, the application of MSE analysis in understanding and optimizing eye movement dynamics in VR opens up new avenues for creating more comfortable and engaging virtual environments. By closely aligning VR design with the natural complexities of human eye movements, we can significantly reduce discomfort and enhance the overall user experience. Future research should continue to explore the relationship between eye movement complexity and user comfort, fostering the development of VR technologies that prioritize user well-being.

### 3.8. Comparative Analysis with Existing Research

Our study contributes to the burgeoning field of VR and eye movement analysis by providing insights into the complexity of gaze patterns in VR environments. Previous research, including Mallari et al.’s systematic review on VR’s impact on chronic pain [34], highlights VR’s potential in enhancing user experiences. Riva et al. [35] and Chiquet et al. [36] have further elucidated the benefits of VR in various therapeutic and wellness contexts, underscoring the versatility of VR applications. Wu et al.’s work [37] on VR in clinical settings adds to the understanding of VR’s applicability across different domains.

Expanding on these foundations, our application of Multiscale Entropy (MSE) analysis to VR gaze data offers a novel perspective on user engagement and cognitive load. Parsons (2015) [38], Kourtesis et al. (2019) [39], and Tashjian et al. (2017) [40] emphasize the importance of immersive and interactive VR experiences for cognitive research and therapeutic interventions. Our findings resonate with these studies by demonstrating how MSE analysis can be used to assess and enhance the VR experience, contributing to a deeper understanding of user behavior in VR environments.

Moreover, recent studies by Tang et al. [41] and Sipatchin et al. [42] highlight the critical role of eye-tracking in VR for cognitive assessments and clinical applications. By integrating MSE analysis with eye-tracking data, our research aligns with the broader trajectory of leveraging VR technology to create immersive, beneficial, and user-centric experiences.

## 4. Conclusions

This study advances our understanding of eye movement behavior in virtual reality (VR) through Multiscale Entropy (MSE) analysis. By dissecting the complexity of eye movements across various VR tasks, we highlight the potential of MSE to contribute to more intuitive and immersive VR environments. The nuanced insights into gaze behavior provided by this analysis are invaluable for enhancing user experience and interface design in VR technology.

### 4.1. Contributions

We conducted a comprehensive investigation into the complexity of eye movement patterns in VR using MSE analysis. Our meticulous analysis of the horizontal and vertical components of eye movements across a range of VR tasks—including vergence, smooth pursuit, video viewing, reading, and random saccades—revealed distinct complexity patterns. These patterns, characterized by logarithmic, logistic, rising, and steady behaviors, contribute to a deeper understanding of gaze predictability and complexity in VR. Our findings offer valuable insights for optimizing VR interfaces and enhancing user experiences by adapting to dynamic user interactions.

### 4.2. Future Work

The continued exploration of MSE and its applications in VR is crucial for unlocking the full potential of this technology for users worldwide. A collaborative approach between academia and industry will accelerate innovation and ensure that VR technology evolves in tandem with our understanding of user needs and preferences. Future research should focus on the following:

- Investigating a broader range of VR tasks and long-term user interactions to provide a more comprehensive understanding of how entropy trends manifest in different contexts.
- Exploring the implications of these findings for enhancing user comfort and engagement in VR, aiming to support the development of more personalized and immersive experiences.
- Integrating complex signal analysis like MSE into VR research, recognizing the potential for transformative impacts on technology and user interaction.

These directions for future research underscore the importance of a nuanced approach to studying VR interactions, with the goal of creating more engaging and sustainable VR environments.

## Data Availability

Data and Python code for reproducing the experiments reported in this article are available upon request. Please contact Sahar Zandi at sahar.zandi@tamu.edu for access.

## Abbreviations

The following abbreviations are used in this manuscript:

MSE	Multiscale Entropy
AR	Augmented Reality
VR	Virtual Reality
VRG	vergence task
VID	video-viewing atsk
PUR	pursuit task
TEX	text-reading task
RAN	random saccades task
